# Detection of Multiple Respiration Patterns Based on 1D SNN from Continuous Human Breathing Signals and the Range Classification Method for Each Respiration Pattern

**DOI:** 10.3390/s23115275

**Published:** 2023-06-01

**Authors:** Jin-Woo Hong, Seong-Hoon Kim, Gi-Tae Han

**Affiliations:** 1 Department of Computer Engineering, Gachon University, Seongnam 13120, Republic of Korea; az13077@gachon.ac.kr; 2 Neowine Co., Ltd., Seongnam 13595, Republic of Korea; shkim@neowine.com

**Keywords:** one-dimensional (1D) CNN, 1D SNN, respiration patterns, MASRP, mmWave sensor

## Abstract

Human respiratory information is being used as an important source of biometric information that can enable the analysis of health status in the healthcare domain. The analysis of the frequency or duration of a specific respiration pattern and the classification of respiration patterns in the corresponding section for a certain period of time are important for the utilization of respiratory information in various ways. Existing methods require window slide processing to classify sections for each respiration pattern from the breathing data for a certain time period. In this case, when multiple respiration patterns exist within one window, the recognition rate can be lowered. To solve this problem, a 1D Siamese neural network (SNN)-based human respiration pattern detection model and a merge-and-split algorithm for the classification of multiple respiration patterns in each region for all respiration sections are proposed in this study. When calculating the accuracy based on intersection over union (IOU) for the respiration range classification result for each pattern, the accuracy was found to be improved by approximately 19.3% compared with the existing deep neural network (DNN) and 12.4% compared with a 1D convolutional neural network (CNN). The accuracy of detection based on the simple respiration pattern was approximately 14.5% higher than that of the DNN and 5.3% higher than that of the 1D CNN.

## 1. Introduction

Respiratory information of humans is being utilized not only for disease diagnosis, but also in various fields related to health, such as healthcare. Several studies are being conducted, including management of bronchiectasis, diagnosis of sleep apnea, analysis of respiration movements, and research on COVID-19’s respiratory symptoms. These studies aim to improve diagnosis, treatment, and management methods in the medical field through the collection, analysis, and interpretation of breathing data [1,2,3,4]. To obtain meaningful respiratory information, recognition and analysis of the respiration patterns from the breathing signal data collected through the measuring device are necessary [5]. Traditionally, a belt-type measuring device with a sensor attached is used to collect breathing signals. However, in this study, breathing data were collected using a non-contact mmWave sensor. Non-contact respiration measurement has the advantage of providing accurate breathing data while increasing patient convenience and safety, and it is used in various medical and research fields [6,7,8,9,10].

The mmWave-sensor-based non-contact method may have lower accuracy than traditional contact-type sensors when noise is inserted due to the existence of an obstacle between the sensor and the body, a change in posture, or the influence of the surrounding environment. On the other hand, the contact sensor-based method attaches the sensor to the body, so it can cause insomnia or sleep disorders, which can cause errors in the acquisition of breathing data. Therefore, to address this problem, studies on artificial-intelligence-based pattern recognition techniques—such as deep neural networks (DNNs) and 1D convolutional neural networks (CNNs)—have been conducted to classify respiration patterns using deep learning methods for breathing data acquired from non-contact sensors [11,12,13].

Previous pattern recognition studies for sleep apnea and other respiration patterns have used data with a fixed input size [14,15]. Recently, various types of information on respiration have been used in the field of healthcare and health examination. Therefore, not only the detection of simple respiration patterns, but also recognition and classification techniques for various respiration patterns—such as the continuous range of the homogeneous respiration pattern, or the ratio of specific respiration patterns in the entire respiration—are required.

In particular, a sliding window method should be used to detect respiration patterns learned from the breathing data measured for a long time period via conventional methods. Interference between different respiration patterns, increased analysis complexity, and signal ambiguity can decrease the accuracy of recognition in respiration translation systems, particularly when multiple patterns are present in one window [16,17]. In other words, one specific respiration pattern must be found within the range of the search unit (one window), but if several respiration patterns exist within the range, it may be misrecognized as another respiration pattern and reduce the overall recognition rate. Furthermore, human respiration patterns are categorized based on the features of respiration cycles and are displayed in various forms, making it difficult to define them with simple threshold values. Even if it is the same respiration pattern, each person may have slightly different respiration patterns.

To solve this problem, this paper proposes a 1D Siamese neural network (SNN) model that can detect specific respiration patterns by comparing the similarity between the input respiration pattern and the basic respiration pattern, along with a novel algorithm that merges consecutive isomorphic pattern ranges and divides other consecutive pattern ranges when there are multiple respiration patterns in the detection target area.

The SNN model is one of the few-shot learning methods that can learn from only a small amount of data and exhibits the advantage of being able to measure the similarity of classes that are not participants in learning [18,19]. To apply this to respiration pattern recognition, the existing SNN structure is used, and all layers for 2D data processing are composed of 1D data processing layers. In the proposed method, the input size of the 1D SNN model is divided into four types (600, 300, 200, and 100) to improve the recognition rate when multiple breathing patterns exist within the search range. This serves to merge or separate the front and rear ranges by applying MASRP while reducing the search range when multiple breathing patterns exist within the search range.

The proposed method improved the classification accuracy of respiration patterns that did not participate in learning. Furthermore, even when changes in respiration patterns within a continuous breathing period were observed, accurate detection of the range was feasible through the application of the proposed merge-and-split for respiration pattern (MASRP) algorithm. The proposed method exhibits substantial accuracy improvement in range classification and respiration pattern detection, as per the experimental observations.

## 2. Related Research

### 2.1. Breathing Signal

**Figure 1:**
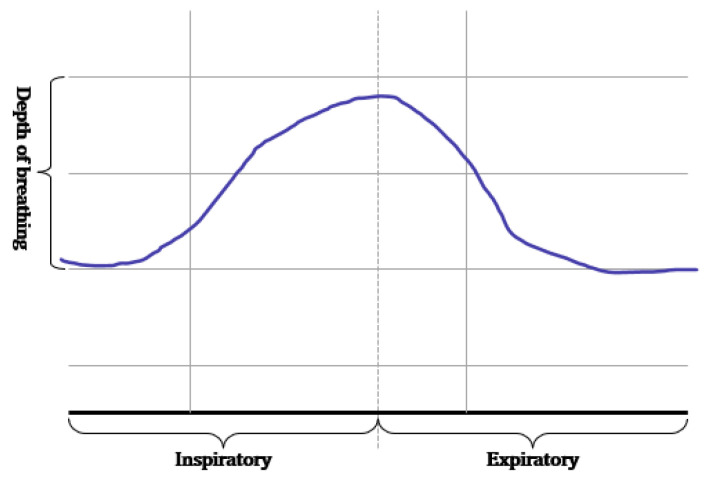
Signals generated according to inhalation, exhalation, and the depth of breathing.

As shown in Figure 1, the breathing signal rises during inhalation and descends during exhalation. The amplitude of the breathing signal is determined by the depth of the breath. Furthermore, the period of the signal increases with the duration of the breath [20].

**Figure 2:**
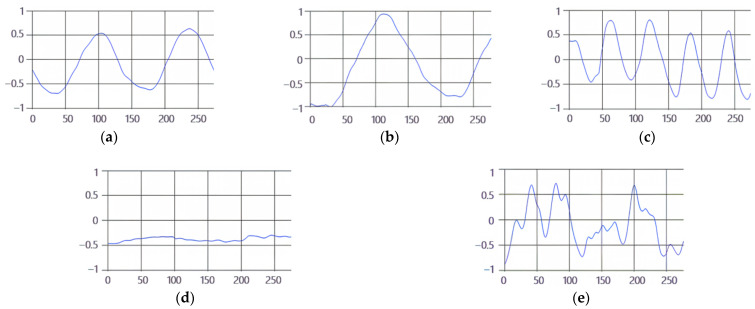
Types of respiration measured by the mmWave sensor: (**a**) eupnea, (**b**) bradypnea, (**c**) tachypnea, (**d**) apnea, (**e**) movement.

These breathing signals appear in various forms depending on the type of breath, and in the medical field they are divided into eupnea, bradypnea, tachypnea, and apnea, based on the number of breaths per minute [21]. The results measured by the mmWave sensor for four respiration patterns and one movement state are shown in Figure 2.

### 2.2. Pattern Recognition Methods of Signal Data Based on Deep Learning

#### 2.2.1. Deep Neural Network (DNN)

A deep neural network (DNN) refers to a neural network composed of two or more hidden layers based on the concept of a multilayer perceptron (MLP). Each layer of the DNN is composed of a defined number of neurons (or nodes), and all neurons are fully connected to one another [22,23,24,25]. DNN learning is a process of updating the weights between connected neurons and is performed by minimizing the value of the loss function through gradient descent [26]. In the case of an image, the input size is in the form of h × w; however, the input size for the 1D signal is configured in the form of 1 × w.

#### 2.2.2. One-Dimensional (1D) Convolutional Neural Network (CNN)

In general, a convolutional neural network (CNN) is a neural network that has been studied for object detection or recognition based on image data. The convolutional layer uses an N × M 2D kernel to extract image features. The features extracted through the convolution of several layers are finally connected to the fully connected layer to perform classification [27].

Since the breathing signal is composed of a one-dimensional signal rather than an image, the two-dimensional image data are transformed to learn them, based on a CNN-based neural network. However, in this case, meaningless features may be generated due to the forced data transformation. This may lead to deterioration in the performance of the neural network. Therefore, constructing a convolutional neural network suitable for 1D data characteristics is necessary [28,29,30,31,32].

In the 1D CNN for learning 1D data, the convolution layer receives a vector type as an input and performs convolution using a 1 × m kernel. Because of this, the feature map is also extracted in the form of one-dimensional data. Therefore, it can be used for learning the breathing signal data. The process of extraction of a feature map by the convolutional layer of a 1D CNN for the classification of the input signal data is similar to the process employed by the convolutional layer of a 2D CNN. Next, the features that undergo the max-pooling process are composed of a fully connected layer for classification, and the shape is the same as that of a 2D CNN [33,34,35]. Appropriately setting hyperparameters such as the depth of the neural network and the size and number of kernels according to the characteristics of the input data is important. Therefore, the process of optimizing hyperparameters using an optimization algorithm is crucial.

#### 2.2.3. Siamese Neural Network (SNN)

**Figure 3:**
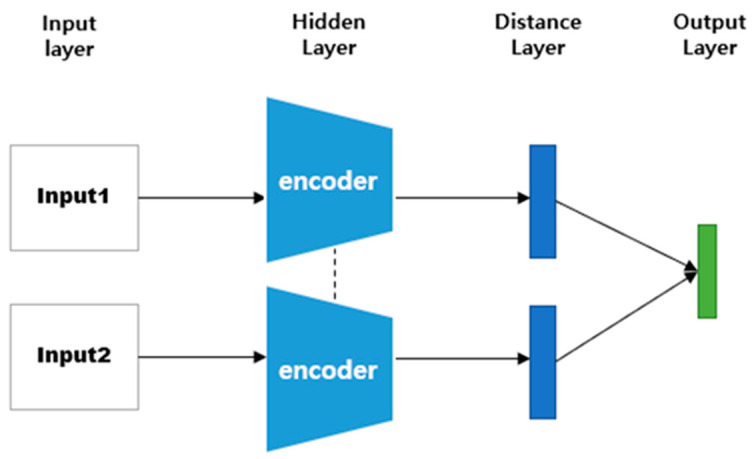
SNN architecture.

A Siamese neural network (SNN) is a neural network composed of two identical CNN-based networks with exactly the same parameters and weights. This model is used in environments where it is difficult to obtain sufficient data for a particular class, or when objects that the model has not learned must be classified [18,36,37,38]. The SNN-based method can predict the class to which a small or once-seen object belongs by using a model trained on other data. To solve these environmental problems, one-shot learning or few-shot learning has been employed [39,40,41,42]. As shown in Figure 3, this model calculates and compares vectors by applying the same weight to two input images. When training is performed, if the two input images are the same, the similarity is assigned as 1; if they are different, it is assigned as 0.

#### 2.2.4. Distance Function

When classifying or recognizing objects, many deep learning models need to be able to distinguish similarities and differences between input objects. The similarities and differences between these objects are abstracted into the concept of distance. In other words, in case of similarity between objects, the distance is close, whereas if there are many differences, the distance is long. A distance function is a function that considers two vectors as inputs and calculates the distance between two points [43,44]. Applying these distance functions according to the characteristics of the data is important for model performance. Furthermore, the distances in the same class are short, whereas the distances in different classes are long.

**Figure 4:**
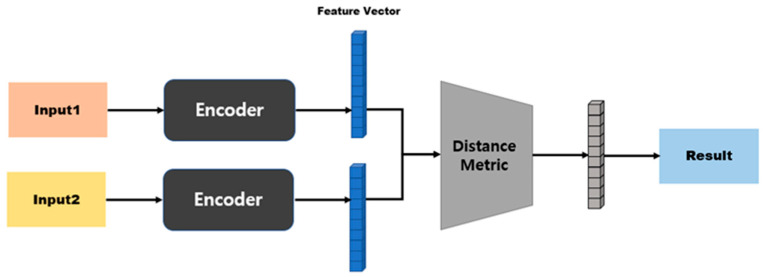
Model structure based on distance metrics.

**Figure 5:**
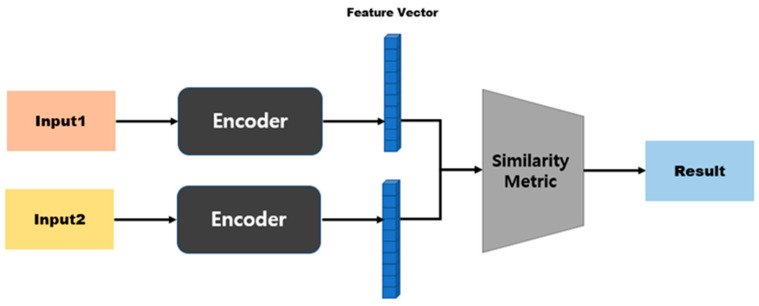
Model structure based on similarity metrics.

In an SNN, the model structure differs depending on the distance function that is used to calculate the distance between two embedding vectors [45,46]. Figure 4 shows the model structure when a distance-based metric is used. Distance-based metrics include Euclidean distance, Manhattan distance, and Hamming distance [47,48]. This model structure maps similarity based on a fully connected layer, because the size of the input vector and the size of the output vector are the same. Figure 5 shows the model structure when a similarity-based metric is used. Since this model structure uses cosine similarity or dot-product similarity, the size of the output vector is always 1, regardless of the size of the input vector; therefore, this structure does not require separate mapping [49,50].

## 3. Proposed Method for the Multiple Respiration Patterns and Each Pattern’s Range Classification Based on a 1D SNN

The existing 1D-CNN-based respiration pattern detection method exhibits suitable performance in detecting one respiration pattern for each type based on the breathing data. However, if there are multiple respiration patterns in the continuous breathing data, classifying and analyzing the ranges for each respiration pattern is difficult, and classification errors may exist. To solve these problems in this study, we propose a 1D-SNN-based respiration pattern recognition model that can be applied even when learning data collection and detection of objects that do not participate in learning are difficult. The reason for approaching the respiration pattern classification method with a 1D SNN model is based on the idea that the most similar respiration pattern can be classified as a target by comparing the similarity between the feature-embedding vectors of the target data and the feature-embedding vectors of the query data. In addition, in the pattern detection result for continuous breathing data, we propose a merge-and-split for respiration pattern (MASRP) algorithm that merges if the contact area is the same pattern and separates it in case of a different pattern.

### 3.1. Limitations of Existing Research

**Figure 6:**
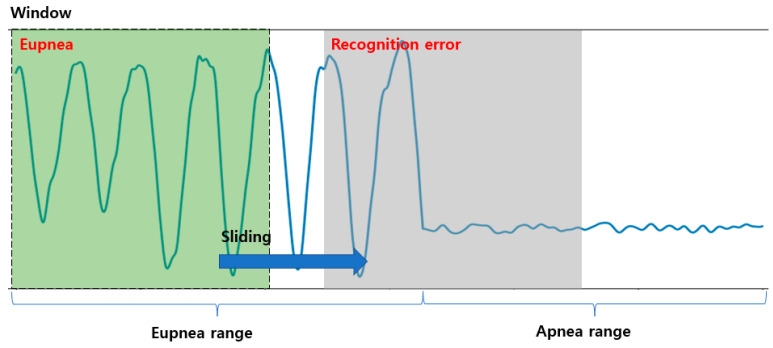
Example of detection of a region with multiple respiration patterns using a 1D CNN and the sliding window method.

DNNs (which are traditional machine learning algorithms) and the recent 1D-CNN-based respiration pattern recognition methods are valid recognition methods when classifying one respiration pattern per input datum. However, if two or more respiration patterns are included in the input data, the recognition rate may be reduced, and results that are not included in the ground truth may appear due to the threshold value. Owing to this problem, when dividing a range by the respiration pattern in the breathing data of a certain range, it can be located in a range containing multiple patterns during the sliding window search, as shown in Figure 6. Figure 6 exhibits an erroneous recognition result between the eupnea and apnea sections. Therefore, the section classification for each respiration pattern may appear as an erroneous result.

Table 1 shows the results of recognizing data containing two respiration patterns in one section using a 1D CNN. Here, even if the two patterns representing the highest probability values are used as classification results, they appear as different results from the actual ground truth. Cases due to recognition errors are displayed in red in Table 1.

### 3.2. Our Proposed Method

**Figure 7:**
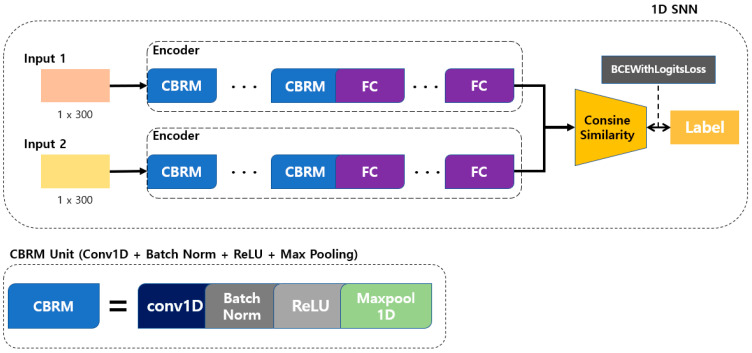
Proposed 1D SNN training model for respiration pattern retrieval.

The structure of the 1D SNN that we propose for multi-respiration-pattern recognition is based on the Siamese network structure. It is modified to allow 1D data to be input to certain layers to process signal data rather than images. The employed distance metrics include the cosine similarity and Manhattan distance. A method with high accuracy is selected for determining the similarity of breathing signal data. The proposed model structure is shown in Figure 7. Breathing data arrive at a rate of 20 data/s through the mmWave radar sensor, and a model with an input size is developed to achieve the best performance (the model with the highest accuracy is a structure with a cosine similarity distance matric and three CBRM blocks with an input size of 300 in 15 s).

As shown in Figure 7, the convolution + batch normalization + ReLU + max pooling (CBRM) unit is composed of a 1D convolution layer, batch normalization, ReLU function, and max-pooling layer. To determine the optimal depth for each input datum size, CBRM units were configured from 2 to 5, and each experiment was conducted accordingly.

In the learning process, the degree of similarity is determined by using cosine similarity for two different feature vectors obtained by inputting two input data to the encoder of the proposed model. The model is trained by inputting the calculated similarity and label values to the BCEWithLogitsLoss function. At this time, the label value is 1 for the same type of breathing and 0 for different types of breathing. BCEWithLogitsLoss is a combination of a sigmoid layer and binary cross-entropy loss, as shown in Equation (1) [51,52,53]. Here, *x* represents the label value, *y* represents the model prediction value, *N* represents the batch size, and *w* represents the weight.

(1) $$\ell \left(x,y\right)=L={\left\{l_{1},...,l_{N}\right\}}^{T},l_{n}=-w_{n}\left[y_{n}\cdot \log\sigma \left(x_{n}\right)+\left(1-y_{n}\right)\cdot \log\left(1-\sigma \left(x_{n}\right)\right)\right]$$

The proposed 1D SNN model consists of Conv1D and MaxPool1D for processing one-dimensional data, as shown in Figure 7. The size of the input, the kernel size of the convolution layer, and the number of kernels are determined through additional experiments, and the optimization of the kernel size uses the harmony search algorithm proposed in the existing 1D-CNN-based respiration pattern recognition research [54]. The neural network architecture of the 1D SNN is constructed using these 1D CNN parameters by combining two 1D CNN models of types A and B. Table 2 shows the structure of the proposed human multiple respiration pattern recognition model.

Table 3 and Table 4 show the DNN and 1D CNN models, respectively, that were applied to detect the existing respiration patterns.

**Figure 8:**
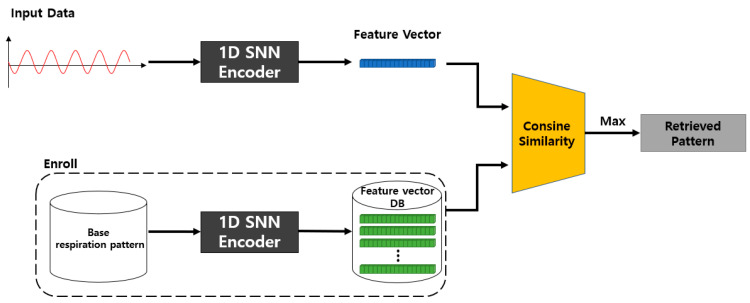
Proposed 1D SNN inference model.

The inference process of the proposed 1D SNN model is shown in Figure 8. Five basic respiration patterns (non-augmented respiration patterns) are input into the previously proposed model, and the resulting feature vectors are all stored in the database. For inference, the feature vector is obtained by inputting the input data for query to the learned model. Subsequently, it calculates the cosine similarity using the feature vector for the query and each feature vector previously stored in the database, and then it returns the respiration pattern with the highest similarity as the detection result.

The 1D-SNN-based respiration pattern learning model (T) proposed in Figure 7 is trained using a fixed-length (m × 1) breathing dataset. The learning model takes two inputs as pairs, and the learning process classifies five basic respiration patterns using cosine similarity and the BCEWithLogitsLoss loss function on the feature-embedding vector of the input data. For model training, the similarity between the same types of respiration patterns is set to 1, and the similarity between different types of respiration patterns is set to 0.

As shown in Figure 8, the proposed SNN-based inference model calculates the cosine similarity between the embedding vectors (B1, …, B5) for each of the five basic respiration patterns and the embedding vector (R) of the query data of fixed length (m × 1) passed through the same model T, trained by the learning model T. The respiration pattern of the query data is detected by selecting the embedding vector from {B1, …, B5} whose similarity with the embedding vector R of the query data is the highest. This method determines which of the five respiration patterns is most similar to R.

Among the hyperparameters of the proposed 1D SNN, the items that influence the recognition accuracy of respiration patterns include the input size, number of layers, and distance metric. The hyperparameters of the 1D CNN constituting the 1D SNN were designed based on the size and number of kernels obtained through previous 1D-CNN-based respiration pattern recognition research [55,56,57]. The distance metric was selected as the result of calculating the accuracy by applying the Manhattan distance and cosine similarity methods to the same model.

**Figure 9:**
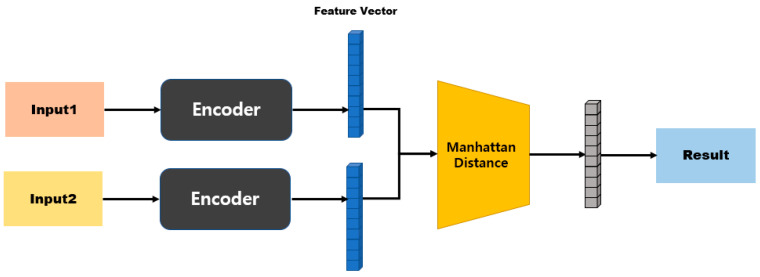
Manhattan-distance-based model structure.

**Figure 10:**
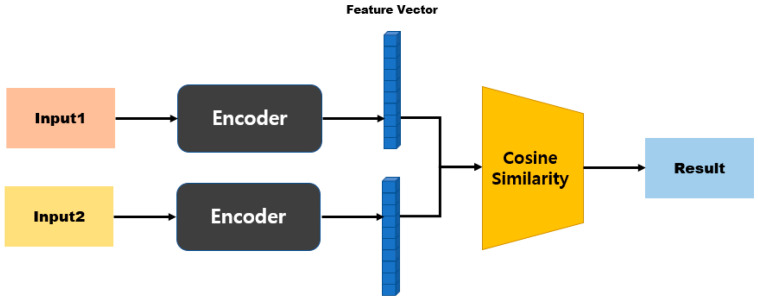
Cosine-similarity-based model structure.

Figure 9 is a model structure based on Manhattan distance, and after passing through the first fully connected layer it exhibits an N-dimensional vector. When two inputs are provided here, the distance can be obtained using Equation (2) for the two outputs. Subsequently, the similarity is measured by processing the last fully connected layer. Figure 10 shows a model structure based on cosine similarity, and after processing the final fully connected layer, an N-dimensional vector is obtained. When two inputs (A and B) are provided, the similarity is obtained using Equation (3).

(2) $$\mathrm{Manhattan}\;\mathrm{Distance}=d\left(A,B\right)=\overset{n}{\underset{i=1}{\sum }}\left|a_{i}-b_{i}\right|$$

(3) Cosine similarity:=Sc(A,B)=cos(θ)=(A⋅B)/‖A‖‖B‖ 

The model configuration to determine the optimized architecture of the proposed model is configured as shown in Table 5. In the model architecture, the layer implies the number of 1D CBRMs, and it is composed of 2–5 for each input size. When designing the model, the minimum kernel size of the 1D convolution layer was set to 6, and when determining the number of layers, the size of the flattened feature vector was set to 64 or more after all of the input data had passed through the CBRM unit. The number of FC layers was configured according to the size of the flattened vector: four if the size was 1024 or more, three if the size was 512 or more, and two if the size was 64 or more. Output dim refers to the size of the feature vector (output dim = 32) after passing all of the layers before cosine similarity calculation.

### 3.3. Merging and Splitting Method of the Respiration Pattern Retrieval Range

**Figure 11:**
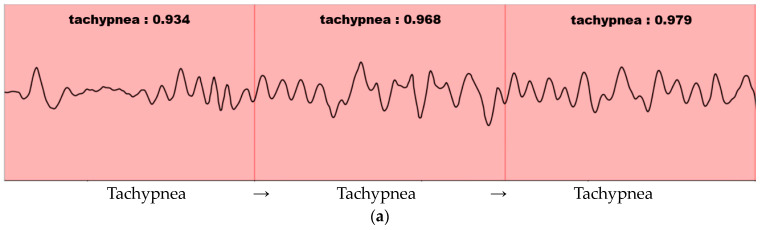

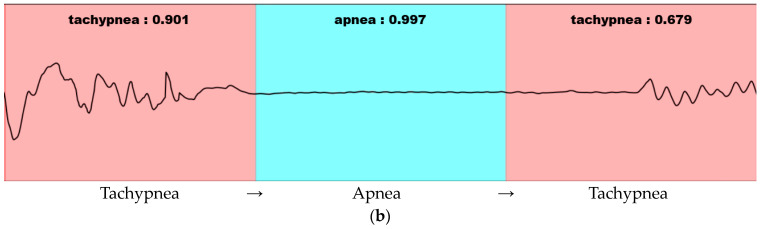
Visualization of 1D-SNN-based respiration pattern recognition results in a continuous breathing range: (**a**) Detection result in the range where the respiration pattern does not change. (**b**) Detection result in the range where the respiration pattern changes. Pink indicates tachypnea and blue indicates apnea.

In general, since the image consists of a background and an object, the objectness score and classification probability are high only at the location of the learned object. Furthermore, since the breathing data consist of continuous patterns learned differently from the image, the probability values of the objectness score and classification are high in almost all locations. The sliding window method is used when detecting the continuous respiration pattern range using a 1D SNN. The stride is set to be the same as the input size. As per the results of the visualized output, several results are output within one continuous respiration pattern range, as shown in Figure 11.

In Figure 11, the detection result is labeled as “Respiration type: Result”. The respiration type represents one of the five respiration patterns (eupnea, bradypnea, tachypnea, apnea, and movement). The result represents the degree of similarity. As shown in Figure 11b, the similarity decreases in the presence of multiple respiration patterns. If the similarity does not exceed the threshold value (set at 0.8 based on the experiments in this study), merging and splitting are performed by applying the merge-and-split for respiration pattern (MASRP) algorithm.

When there are two or more respiration patterns in the target area for respiration pattern detection, MASRP connects the same respiration pattern area in the front and back with the same respiration pattern. It separates the area when there are different types of respiration patterns in the front and back. The purpose of this process is to determine the stability and instability of breathing. This is achieved by calculating consecutive sections of the same respiration pattern and determining the number of specific respiration patterns and the length of each respiration pattern within the entire breathing section.

**Figure 12:**
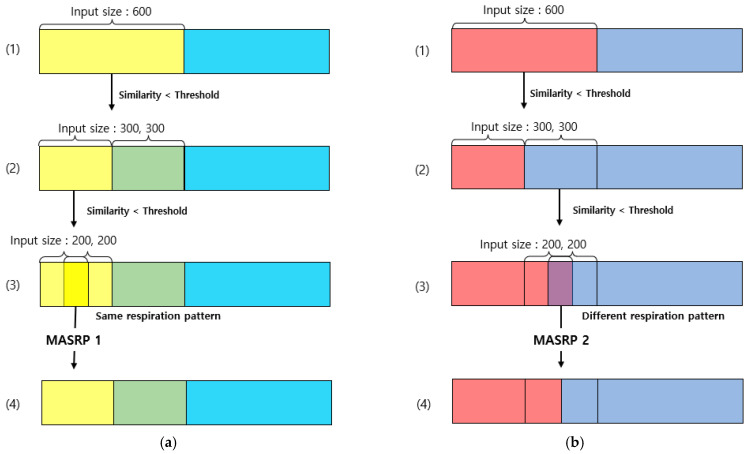
Examples of merging and splitting performed on the respiration pattern recognition results of the 1D SNN: (**a**) Process of applying MASRP 1. (**b**) Process of applying MASRP 2. Each color is a respiration pattern, and (1)–(4) indicate the stages of merging and splitting.

Each respiration pattern is displayed by matching colors in Figure 12. Eupnea is expressed in blue, bradypnea in yellow, tachypnea in red, apnea in cyan, and movement in green. To detect a section of a continuous respiration pattern, the corresponding respiration pattern is classified based on the procedure shown in Figure 12, and the MASRP algorithm is applied differently depending on whether the continuous respiration pattern is the same or different. To detect continuous breathing sections, as shown in Figure 12, the first input is used to detect respiration patterns in units of 600 (30 s).

Considering the execution procedure, Figure 12a presents the process of MASRP 1, and Figure 12b presents the process of MASRP 2. In Figure 12a, (1) presents the similarity values and respiration patterns based on the inputs presented to the 1D SNN model in units of 600 (30 s) from the input breathing data. The (2) in panel (a) shows the similarity value and respiration pattern found after inputting the data divided into 300 (15 s) units to the proposed 1D SNN model for the range where the similarity value in (1) is lower than the threshold. For the range in which the similarity value is lower than the threshold in (2), the range in (3) is divided into 200 (10 s) units and is inputted to the proposed 1D SNN model. In (3) in panel (a), if the two respiration patterns are the same, Algorithm 1 is employed to merge the corresponding areas into one area; (4) shows the result of executing Algorithm 1.

In Figure 12b, (1) shows the division of the input data size by 300 when the similarity obtained by using a 1D SNN model for respiration pattern recognition with an input data size of 600 falls below a certain threshold (in this case, 80); (2) shows the similarity obtained by inputting the divided data into a 1D SNN model with an input data size of 300 and dividing the input size by 200 when the similarity falls below a certain threshold. When dividing by 200, the division is based on 200 from the starting point and the ending point, and a 100-sized respiration pattern overlaps; (3) uses a 1D SNN model with an input size of 200 to obtain a 200-sized respiration pattern. If the two inputs have the same respiration pattern, applying the merging algorithm MASRP 1 yields the result shown in (4) in panel (a). However, if they have different respiration patterns, applying the partitioning algorithm MASRP 2 yields the result shown in (4) in panel (b). In this case, the left and right areas are divided according to the similarity ratio of the two respiration patterns.

**Figure 13:**
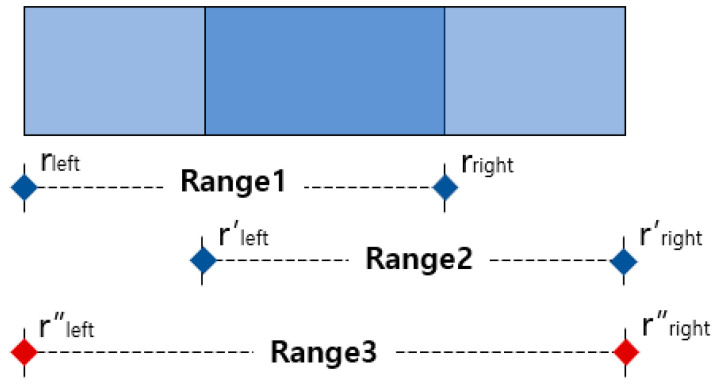
Example of application of an algorithm that merges overlapping ranges of the same pattern. The red dot indicates the range of merged result.

**Figure 14:**
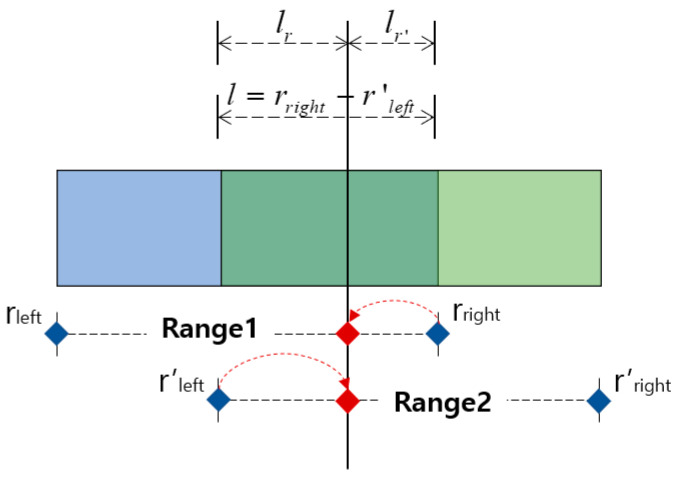
Example of the splitting algorithm’s performance when two different patterns overlap one another as a result of detection. The red dot indicates the range of split result.

Figure 13 describes Algorithm 1 (MASRP 1). Furthermore, as with (3) in Figure 12b, if the two respiration patterns are different, the overlapping range is cut into units of 100 (5 s), input to the proposed 1D SNN model, and divided into two ranges using Algorithm 2. Furthermore, the division criterion is the ratio of similarity of each respiration pattern existing in the range. Figure 14 describes Algorithm 2 (MASRP 2).
**Algorithm 1 (MASRP 1):** Algorithm to merge two ranges of the same overlapping pattern
①Assign r_left_ of range 1 to r″_left_ of range 3.r″_left_ = r_left_②Assign r′_right_ of range 2 to r″_right_ of range 3.r″_right_ = r′_right_③Get range 3 as a result of merging range 1 and range 2.

The MASRP 1 algorithm merges two overlapping regions (range 1 and range 2) detected by the 1D SNN model when they have the same respiration pattern. During the merge process, as shown in ①, the r_left_ of range 1 is assigned to the r″_left_ of range 3, and the r′_right_ of range 2 is assigned to the r″_right_ of range 3, as shown in ②. The merged result of range 1 and range 2 is represented as range 3, as shown in ③.
**Algorithm 2 (MASRP 2):** Algorithm to split the region when two ranges r and r’ detected with different patterns overlap one another

①Calculate the length *l* of the overlapping ranges.*l* = r_right_ − r′_left_②After inputting the overlapping range *l* to the proposed 1D SNN model, divide *l* using the similarity value Sc, Sc′ (Sc is the similarity with the Range1 class; Sc′ is the similarity with the Range2 class) as a ratio value.*l*_r_ = *l* × {S_c_/(S_c_ + S_c_′)}, *l*_r′_ = *l* × {S_c_′/(S_c_ + S_c_′)}③Adjust r_right_ and r′_left_ using divided *l*_a_ and *l*_a_′.r_right_ = r_right_ − *l*_a_′, r_left_′ = r_left_′ + *l*_a_

MASRP 2 divides two regions based on the similarity ratio of the detected overlapping regions (range 1 and range 2) from the 1D SNN model when they have different respiration patterns. To do this, first, calculate the length *l* of the overlapped range, as in ①, and input it into the 1D SNN model to obtain the similarity Sc with the range l respiration pattern and the similarity Sc′ with the range 2 respiration pattern. Then, divide *l* into *l*_r_ and *l*_r′_ by their respective similarity ratios, as in ②. Finally, as in ③, determine range 1 and range 2 by adjusting the r_right_ of range *l* with *l*_r′_ and the r′_left_ of range 2 with *l*_r_.

## 4. Experimental Results and Considerations

In this section, the breathing data collection environment for the experiment and the composition of the collected data are presented. The experimental results of the proposed 1D SNN respiration pattern classification model and the results of the application of existing methods (such as DNN and CNN) are compared and evaluated. The performance evaluation of the proposed 1D SNN evaluates the accuracy based on IOU by comparing the ground truth with the result of applying the proposed method to test the data on a trained model. 

For comparison with the existing method, a respiration pattern recognition model was implemented with a DNN and a 1D CNN. Subsequently, the results of applying MASRP (DNN + MASRP, 1D CNN + MASRP) and IOU accuracy with the ground truth were calculated. We calculated the IOU between the result of the proposed method (1D SNN + MASRP) and the ground truth. Furthermore, we calculated the IOU between the results of applying MASRP to the existing methods (DNN, 1D CNN) and the ground truth. The proposed method was found to be superior to the existing methods.

### 4.1. Experimental Environment

**Figure 15:**
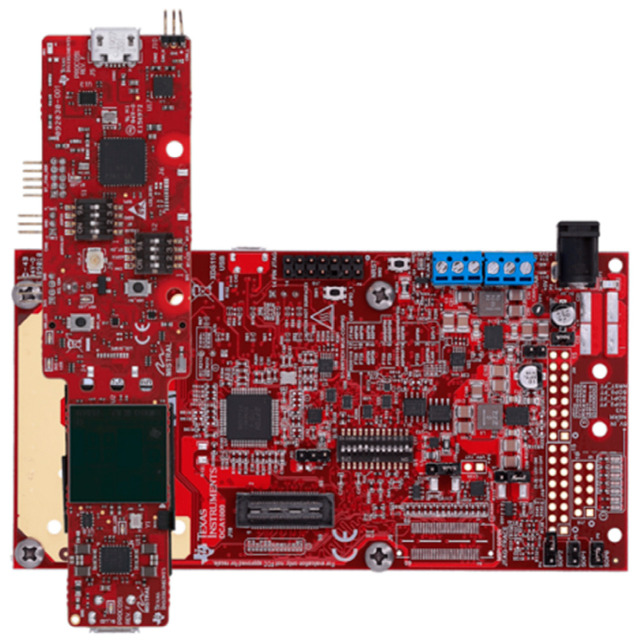
The mmWave sensor used to collect breathing signal data.

The mmWave sensor shown in Figure 15 was used for the experiments in this study, and the specifications of each device are shown in Table 6. Each sensor is composed of an environment that can measure data through PC and UART communication [58].

**Figure 16:**
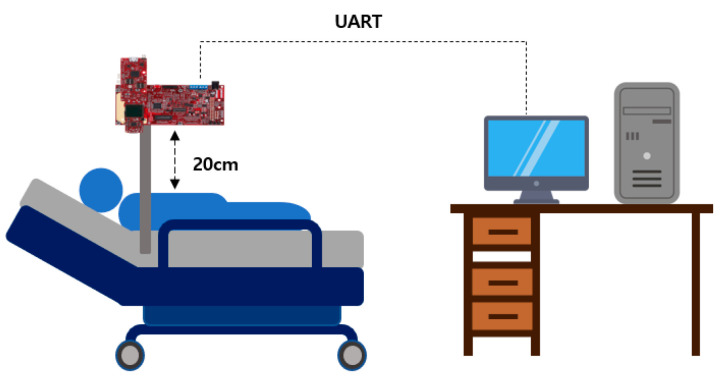
The mmWave sensor installation environment for collecting breathing signal data.

**Figure 17:**
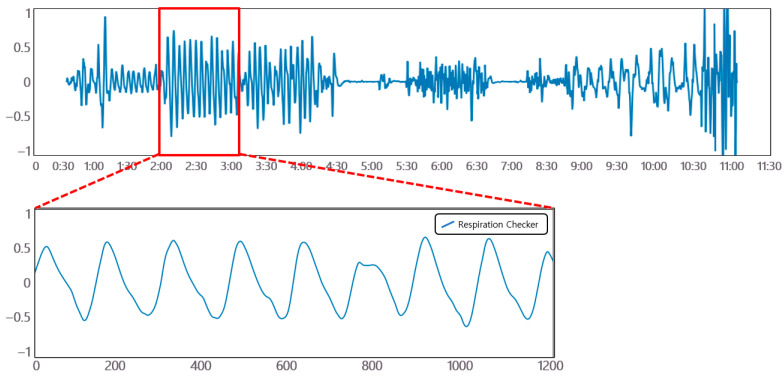
Example of a breathing signal measured with the mmWave sensor.

Pertaining to the breathing signal collection environment using the mmWave sensor, as shown in Figure 16, a person lies in an upright position on a simple bed, and the mmWave sensor is installed at a location 20 cm away from the person’s chest to collect signals for five respiration patterns. Figure 17 shows an example of a breathing signal measured using the mmWave sensor.

**Figure 18:**
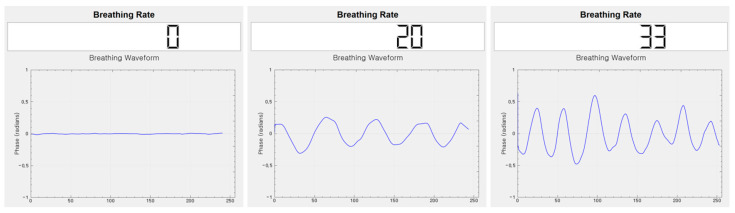
Breathing data collection program using the mmWave sensor.

In the aforementioned environment, data were collected using a breathing data measurement program, as shown in Figure 18. Breathing signal data were collected while inducing eupnea, bradypnea, tachypnea, apnea, and movement signals to be included.

The system environment for learning and testing the collected data was implemented on a PC, as shown in Table 7.

### 4.2. Organization of Training and Test Data

**Figure 19:**
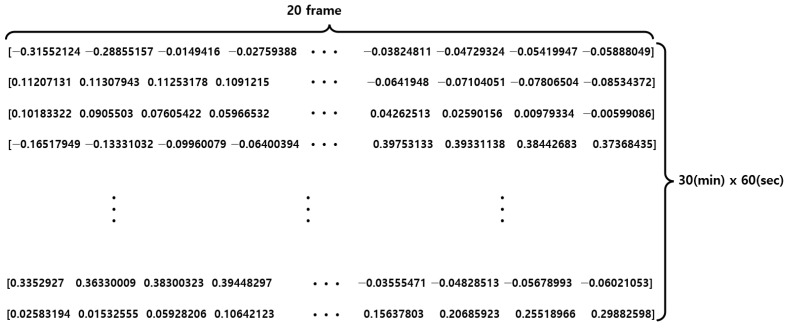
Raw data structure of the mmWave sensor.

For training and testing purposes, a dataset was constructed using mmWave sensors for 10 people, collecting 30 breathing data per person for 30 min each. To prevent diversity and potential bias of data, 250 breathing data were collected from 7 out of 10 individuals for training purposes, while the remaining 50 breathing data were collected from the other 3 individuals for testing purposes. Figure 19 shows the structure of the raw data obtained from the mmWave sensor.

**Figure 20:**
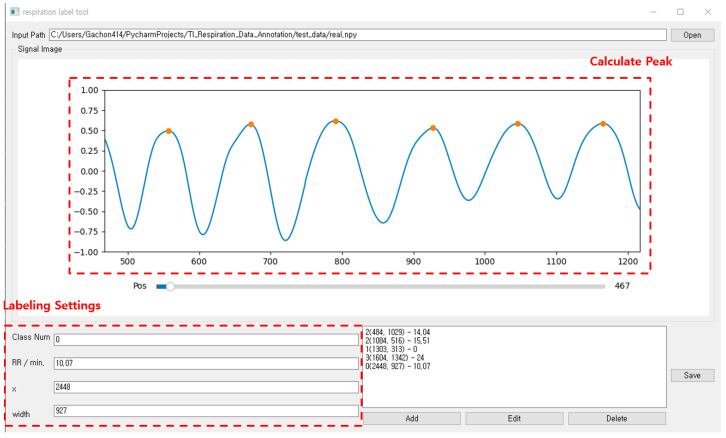
Annotation tool for breathing data pattern area designation and labeling.

As shown in Figure 20, the respiration pattern range can be set via the annotation tool developed for this study. When the range is set, the center point (x) and length (width) information of the range are automatically entered in the labeling setting area. The screen displays the respiration rate per minute for the selected area. The respiration rate per minute is calculated according to the number of peaks in the set area after calculating the peak for the breathing signal. Finally, the ground truth for the respiration pattern area is set by clicking the “add” button after entering the class number information. By repeating this operation, annotation is performed on the continuous breathing data. After all labeling is completed, the save button may be clicked to save the breathing data file (.npy) and the annotation information (.txt) file.

**Figure 21:**
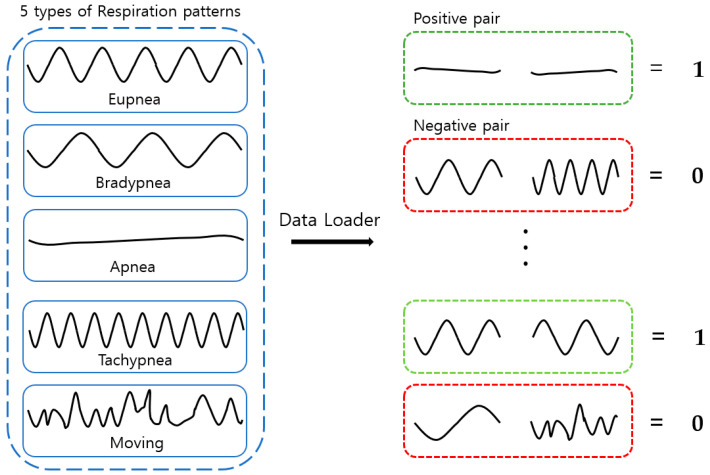
Training and testing data loaders.

The dataset created through annotation is shown in Table 8. The number of respiration patterns and the length of the data according to the ground-truth set are shown. Each breathing datum was cut into 30-minute lengths, and there were a total of 36,000 data points (20 data/s × 60 s × 30 min) within that range. Out of the entire dataset—consisting of 300 breathing data samples with a length of 30 min each—250 were used for training and 50 were used as test data. When configuring the training data loader for pattern classification, one pair of random positive pairs and one pair of random negative pairs that fit the set input size among the 250 training data were imported, as shown in Figure 21. When configuring the test data loader, one pair of random positive pairs and N pairs of random negative pairs were imported that fit the set input size among the 50 test data. In the case of the same respiration pattern, a positive pair features a label value of 1, and in the case of a different respiration pattern, a negative pair features a label value of 0.

### 4.3. Experiment and Evaluation

In this section, we performed 1:1 verification of respiration patterns for each input size and layer depth for the 1D SNN model, i.e., the proposed method. Furthermore, we also evaluated the classification accuracy of respiration patterns for each input size and layer depth. Through the classification of continuous breathing ranges, respiration patterns were first detected by applying the 1D SNN model, and they were subsequently classified into ranges of each respiration pattern based on application of MASRP to the detected areas. The final results of the application of MASRP and the ground truth were interpreted in terms of IOU to evaluate the accuracy of the proposed method.

In addition, the superiority of the proposed method was demonstrated by comparing the IOU between the ground truth and the final result of classification of the respiration pattern area through the application of MASRP to the results of respiration pattern recognition based on the existing DNN and 1D CNN methods.

#### 4.3.1. Pattern Classification Experiments and Results

Experiments were conducted to determine the optimal input size for respiration interval detection of the proposed 1D-SNN-based model. The basic hyperparameters required for learning and testing are defined in Table 9. The basic hyperparameters include test trials, way, and num train. Here, test trials pertain to the number of samples required for comparison when conducting an experiment on the test dataset. Way is a parameter that adjusts the ratio of positive pairs to negative pairs when conducting the trials. Num train is a parameter that determines how many data will be randomly extracted and trained per epoch.

There were four different input data sizes: 100 (5 s), 200 (10 s), 300 (15 s), and 600 (30 s). Twenty pieces of data were collected per second from the mmWave radar sensor. 

Cosine similarity and L1 distance were used as distance metrics, and each layer was configured to have 2–5 layers. For 20 epochs, if the maximum accuracy was not updated, learning was set to stop, and 400 × 5 (test trials × way) random data were imported in one test. The ratio of positive pairs to negative pairs was 1:5 (1:way). The total number of epochs was 200, and the batch size was set to 4096.

Table 10 shows the 1:1 verification accuracy for each metric and input data size. In the experimental results, the overall accuracy of the metric function using cosine similarity was higher than that using the Manhattan distance metric. Considering the accuracy of 1:1 verification, the model structure exhibiting the highest accuracy by input size showed the highest performance when the layer depth was set to three for the input sizes of 100, 300, and 600. At 200, it was slightly higher when the layer depth was four. The results of evaluating the retrieval accuracy with the model are shown in Table 11. The performance was the highest at the input size of 300, with 87.4% at the input size of 100, 92.0% at 200, 97.6% at 300, and 97.4% at 600. In this study, the input data size was sequentially selected as 600, 300, 200, and 100 to detect continuous respiration pattern ranges. At this time, the model with the highest top-1 accuracy was applied for each input data size.

**Figure 22:**
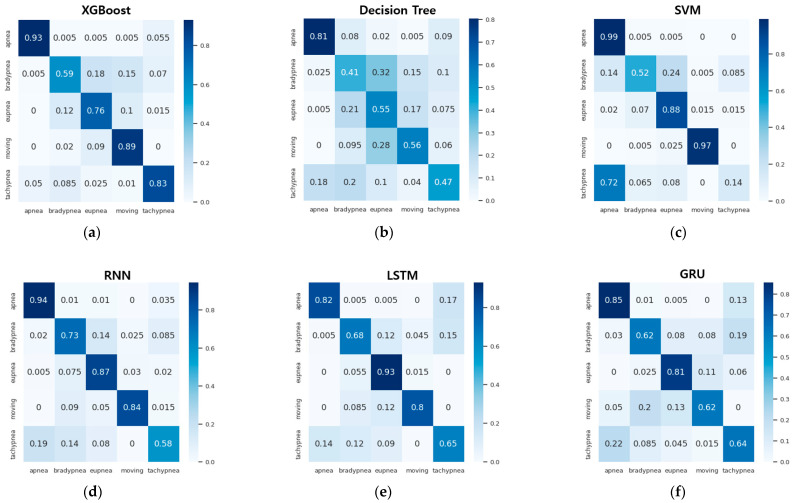

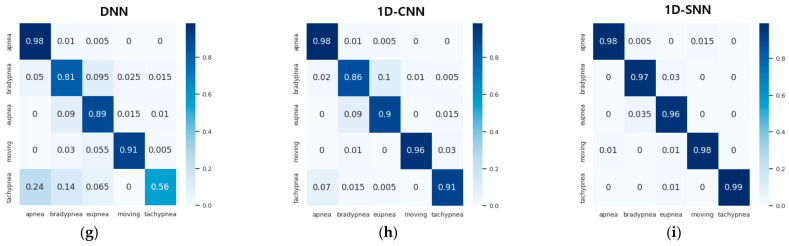
Confusion matrix comparison of pattern recognition results between the proposed method and existing methods: (**a**) XGBoost method, (**b**) decision tree method, (**c**) SVM method, (**d**) RNN method, (**e**) LSTM method, (**f**) GRU method, (**g**) DNN method, (**h**) 1D CNN method, (**i**) 1D SNN method.

Table 12 and Figure 22 show the accuracy and confusion matrix of each respiration pattern for the proposed method (1D-SNN) and existing methods for respiration pattern recognition results. The test data used to measure the accuracy of each respiration pattern consisted of a total of 2000 test datasets, each containing 400 respiration patterns, and the input data size was 300. In addition, Table 13 shows the recall, precision, and F1 scores for the respiration pattern recognition results of the proposed method and existing methods. As shown in Table 12, the experimental results revealed that the 1D SNN, 1D CNN, and DNN methods exhibited high accuracy in order. Therefore, the proposed MASRP algorithm was applied, with a focus on 1D SNN, 1D CNN, and DNN, to improve the classification accuracy of multiple respiration patterns in continuous breathing segments.

#### 4.3.2. Evaluation of IOU accuracy for Merging and Splitting Results of Respiration Pattern Ranges

**Figure 23:**
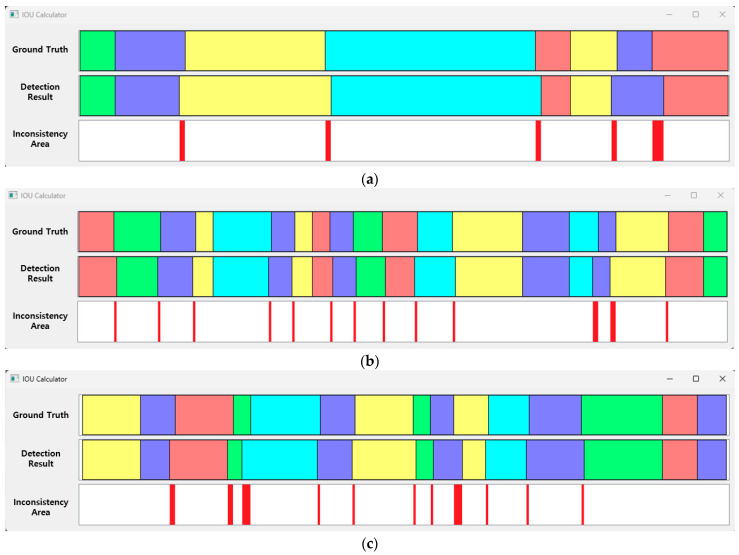
IOU samples between the ground truth of the test data and the detection results of the proposed method (respiration pattern visualization: eupnea = blue, bradypnea = yellow, tachypnea = red, apnea = cyan, movement = green). (**a**) IOU: 94.6%, (**b**) IOU: 93.3%, (**c**) IOU: 92.4%.

For the evaluation of the proposed method, first, a 1D SNN model was used to detect respiration patterns in the test dataset. The range merging and splitting processes were performed using the MASRP 1 and 2 algorithms proposed in Section 3.3 for the detection results. As shown in Figure 23, the ground-truth area of the test data and the results of the proposed method were compared in terms of the IOU across several samples. The error is indicated as the discrepancy area. The results of the ground truth and 1D SNN detection are visually displayed by overlaying colors for each respiration pattern.

Figure 23 shows the ground truth for the test data and the processing results obtained applying the 1D SNN and the MASRP algorithm on the test data. The inconsistency range shows the error between the ground truth and the detection results of the proposed method.

The accuracy of the aforementioned results can be changed according to the ignore threshold value during inference. This value is a threshold value for determining whether the model correctly detected the pattern range. As the value is smaller, multiple detection results may occur in one respiration pattern region, and the probability of false detection increases accordingly. Therefore, in this paper, a relatively high value of 0.8 was acquired, such that an appropriate number of detection results were obtained. If this value is set to be extremely high, holes may appear with a high frequency between the detected pattern areas. Inconsistent areas mostly occurred in the section where the respiration pattern changed. In particular, when the movement pattern area signal was similar to other respiration patterns, it was recognized as a respiration pattern, and the discrepancy was substantial.

**Figure 24:**
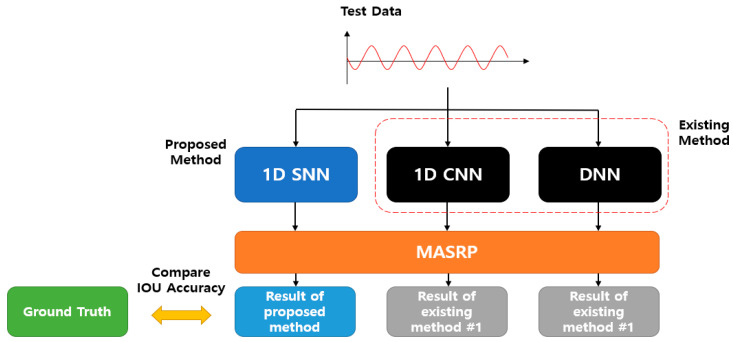
IOU accuracy comparison evaluation method between the existing and proposed methods.

The accuracy comparison of the proposed 1D-SNN-based detection method (1D SNN + MASRP) was conducted using 50 test data, as shown in Figure 24. For accuracy comparison, we calculated the IOU between the results of applying MASRP to the existing methods (1D CNN and DNN) and the ground truth. Thereafter, we calculated the IOU between the result of the proposed method (1D SNN + MASRP) and the ground truth. The method with high IOU exhibited high detection performance. Existing methods such as 1D CNN and DNN use pretrained models.

**Figure 25:**
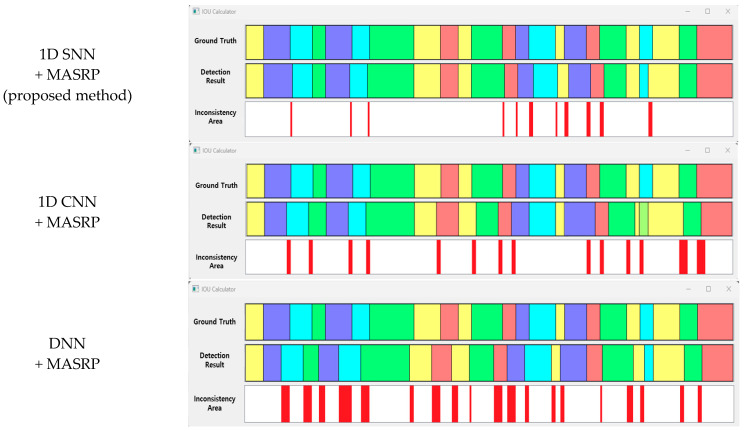
Visualization of samples for IOU accuracy comparison between the existing method and the proposed methods (eupnea: blue, bradypnea: yellow, tachypnea: red, apnea: cyan, movement: green).

The results of applying MASRP to the proposed 1D SNN and the results of applying MASRP to existing methods were visualized, as shown in Figure 25. When the results of each pattern were observed, the proposed method appeared much less frequently than the existing methods in the inconsistent area. The existing methods also encountered the problem of recognizing a part of a specific pattern area as a completely different pattern from the ground truth.

Experiments were conducted on 50 test data, and the average IOU results of the proposed method and the existing methods are shown in Table 14. The proposed method exhibited approximately 12.4% higher accuracy than the 1D CNN method and 19.7% higher accuracy than the DNN method.

## 5. Conclusions

Recently, various studies have been conducted on the analysis of human sleep quality or sleep breathing disorders using artificial intelligence technology. Existing studies have mainly focused on simple classification of apnea patterns. However, recently, in the field of healthcare or sleep-related healthcare, research that recognizes various respiration patterns and the classification of simple respiration patterns has gained considerable interest. In particular, research on recognizing various respiration patterns from long-term continuous breathing data and extracting information such as frequency and duration for each respiration pattern requires attention. In this paper, we propose a neural network model that detects regions for each respiration pattern from long-term breathing data and an algorithm that merges and divides regions into regions where multiple respiration patterns exist. 

The proposed neural network is based on 1D SNN. We designed a 1D SNN model that can detect respiration pattern regions from one-dimensional breathing data. In addition, a study was conducted to extract information such as the frequency and duration of each respiration pattern from the entire breathing dataset by applying a merge-and-split algorithm (MASRP) to the respiration pattern regions detected by the proposed model. In particular, the proposed method improves the performance by optimizing the main hyperparameters of 1D SNN through the harmony search algorithm and additional experiments. Since the 1D SNN classifies respiration patterns based on similarity, registering individual respiration patterns in the model can improve the detection accuracy. In addition, even with long-term breathing data, it is possible to identify the ratio and frequency of each respiration pattern in the data, which can be utilized in the medical field.

The data from 10 normal adults were used for learning and testing. Using the mmWave sensor, 30 cases of 30-minute-long data were measured for each person; that is, a total of 300 data were secured. Each datum was measured to include all five patterns of eupnea, bradypnea, tachypnea, apnea, and movement. To evaluate the performance of the proposed method and the existing respiration pattern recognition methods (1D CNN and DNN), the sliding window method was used to detect the respiration pattern area, and the accuracy of the detection results was compared with the IOU between the result of applying the proposed MASRP algorithm and the ground truth. As a result, the proposed method exhibited approximately 12.4% accuracy improvement over the 1D CNN method and approximately 19.7% accuracy improvement over the DNN method. In other words, the average accuracy of classification by respiration pattern confirmed that the proposed method was improved by approximately 14.5% over DNN and 5.3% over 1D CNN. 

While proposing a classification method for respiration patterns with high accuracy, it is difficult to guarantee 100% accuracy, due to the possibility of multiple respiration patterns within the detection range. Comparing multiple respiration pattern components within the target detection window with the existing respiration patterns makes it difficult to ensure high accuracy. Therefore, we proposed a 1D SNN model based on the similarity of embedding vectors for respiration pattern features in our proposed method, to improve the accuracy. Additionally, we increased the classification accuracy by applying the proposed MASRP algorithm when multiple respiration patterns existed in the detection range. In this case, we divided the detection range into detailed sections and applied the 1D SNN to each section. After division, when multiple respiration patterns existed in the last detailed section, we divided the section into “before and after” respiration ranges based on the similarity ratio of each respiration pattern, and then merged them. Therefore, it is still difficult to guarantee 100% accuracy.

## Figures and Tables

**Table 1:** Error examples from the results retrieved in the respiration range containing multiple patterns (E: eupnea, B: bradypnea, T: tachypnea, A: apnea, M: movement). Error recognition is shown in red, and normal recognition is shown in blue.

Multiple Patterns (Ground Truth)	Input Breathing Signals	Probability Value for Each Pattern Retrieved from the Multiple Respiration Pattern Range				
		E	B	T	A	M
E-A	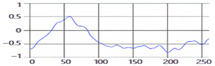	**0.21**	**0.61**	0.02	0.12	0.04
E-M	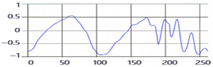	0.18	0.09	**0.27**	0.04	**0.42**
B-T	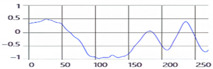	**0.51**	0.1	**0.21**	0.03	0.15
T-A	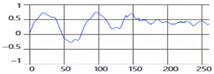	0.04	0.01	**0.63**	0.2	**0.12**
A-M	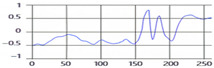	0.02	0.03	**0.21**	0.08	**0.66**

**Table 2:** Proposed 1D SNN architecture for detecting respiration patterns.

Layer	Type A	Filters	Size/Stride	Type B
0	Conv1D	64	20 × 1/1	Conv1D
1	BatchNorm1D	64		BatchNorm1D
2	ReLU			ReLU
3	MaxPool1D		2 × 1/2	MaxPool1D
4	Conv1D	32	20 × 1/1	Conv1D
5	BatchNorm1D	32		BatchNorm1D
6	ReLU			ReLU
7	MaxPool1D		2 × 1/2	MaxPool1D
8	Conv1D	16	20 × 1/1	Conv1D
9	BatchNorm1D	16		BatchNorm1D
10	ReLU			ReLU
11	MaxPool1D		2 × 1/2	MaxPool1D
12	FC			FC
13	FC			FC
14	FC			FC
15	Cosine similarity (embedding vector of type A; embedding vector of type B)			

**Table 3:** Existing DNN architecture for detecting respiration patterns.

Layer	Type	Input Size	Output Size
0	Linear	300	256
1	BatchNorm1D	256	
2	ReLU		
3	Linear	256	128
4	BatchNorm1D	128	
5	ReLU		
6	Linear	128	64
7	BatchNorm1D	64	
8	ReLU		
9	Linear	64	32
10	BatchNorm1D	32	
11	ReLU		
12	Linear	32	5
13	BatchNorm1D	5	
14	ReLU		
15	Softmax	5	5

**Table 4:** Existing 1D CNN architecture for detecting respiration patterns.

Layer	Type A	Filters	Size/Stride
0	Conv1D	256	20 × 1/1
1	BatchNorm1D	256	
2	ReLU		
3	MaxPool1D		2 × 1/2
4	Conv1D	128	20 × 1/1
5	BatchNorm1D	128	
6	ReLU		
7	MaxPool1D		2 × 1/2
8	Conv1D	64	10 × 1/1
9	BatchNorm1D	64	
10	ReLU		
11	MaxPool1D		2 × 1/2
12	Dropout		0.4
13	FC		
14	FC		
15	FC		
16	Softmax		

**Table 5:** Layer configuration diagram according to the input size for the proposed model.

Size	Layer	Architecture							Output Dim.
100	2	CBRM	CBRM	FC	FC	-	-	-	32
	3	CBRM	CBRM	CBRM	FC	FC	-	-	
200	2	CBRM	CBRM	FC	FC	FC	-	-	
	3	CBRM	CBRM	CBRM	FC	FC	-	-	
	4	CBRM	CBRM	CBRM	CBRM	FC	FC	-	
300	2	CBRM	CBRM	FC	FC	FC	FC	-	
	3	CBRM	CBRM	CBRM	FC	FC	FC	-	
	4	CBRM	CBRM	CBRM	CBRM	FC	FC	-	
600	2	CBRM	CBRM	FC	FC	FC	-	-	
	3	CBRM	CBRM	CBRM	FC	FC	FC	FC	
	4	CBRM	CBRM	CBRM	CBRM	FC	FC	FC	
	5	CBRM	CBRM	CBRM	CBRM	CBRM	FC	FC	

**Table 6:** The mmWave sensor specifications for breathing data measurement.

Item	Specification
Maximum detection range	20 m
Frequency range	60–64 GHz
Elevation FOV	±60°
Gain	5 dBi
Azimuth FOV	±60°

**Table 7:** Learning and testing system environment of the proposed 1D SNN.

Item	Specification
CPU	Intel Core i7-10700K
GPU	NVIDIA GeForce RTX 3090Ti
RAM	64 GB
O/S	Ubuntu20.04
Deep learning framework	PyTorch
Program language	Python3.8
CUDA/CuDNN	11.3/8.1.3
IDE	PyCharm
Other libraries	PyQT5, Python-Opencv

**Table 8:** Results of annotation based on the ground truth for each breathing dataset.

No. of Data	Data Length	Number of E Pattern	Number of T Patterns	Number of B Patterns	Number of A Patterns	Number of M Patterns
1	20 (data) × 60 (s) × 30 (min) = 36,000	6	3	2	3	5
2		3	3	1	2	1
3		6	3	3	1	2
4		5	2	3	1	1
5		5	2	4	2	1
...		...				
298		7	2	3	5	2
299		5	6	6	4	4
300		4	3	4	4	6

(E: eupnea, T: tachypnea, B: bradypnea, A: apnea, M: movement).

**Table 9:** Definition of SNN hyperparameters.

Hyperparameters	Definition	Defined Parameters
Test trials	Test one-shot trials	400
Way	Ways in the one-shot trials	5
Num train	Respiration values in training dataset	90,000
Batch size	Respiration values in each batch of data	4096
Num workers	Sub-processes to use for data loading	4
Shuffle	Whether to shuffle the dataset between epochs	True
Epochs	Epochs to train	200
Init momentum	Initial layer-wise momentum value	0.5
Lr patience	Number of epochs to wait before reducing lr	1
Train patience	Number of epochs to wait before stopping train	20

**Table 10:** The 1:1 verification accuracy per input data size and metric.

Input Data Size	Metric	Layer	Accuracy
100	Cosine similarity	2	82.19
		3	82.81
	Manhattan distance	2	71.56
		3	81.56
200	Cosine similarity	2	91.56
		3	90.94
		4	92.50
	Manhattan distance	2	80.63
		3	82.81
		4	84.06
300	Cosine similarity	2	96.56
		3	98.44
		4	93.44
	Manhattan distance	2	80.63
		3	88.13
		4	86.56
600	Cosine similarity	2	97.81
		3	98.05
		4	96.56
		5	95.31
	Manhattan distance	2	56.88
		3	66.13
		4	89.06
		5	86.56

**Table 11:** Top-1 accuracy for respiration pattern detection according to the input size and layer depth.

Input Data Size	Layer	Accuracy
100	2	87.4
	3	84.2
200	2	91.6
	3	92.0
	4	91.8
300	2	95.6
	3	97.6
	4	91.8
600	2	97.0
	3	97.4
	4	96.2
	5	95.4

**Table 12:** Comparison of accuracy for each respiration pattern between the proposed method and existing methods.

Method	Apnea	Bradypnea	Eupnea	Movement	Tachypnea	Avg. (%)
XGBoost	0.93	0.59	0.76	0.89	0.83	80.1
Decision tree	0.81	0.41	0.55	0.56	0.47	56.3
SVM	0.99	0.52	0.88	0.97	0.14	69.9
RNN	0.94	0.73	0.87	0.84	0.58	79.5
LSTM	0.82	0.68	0.93	0.8	0.65	77.5
GRU	0.85	0.62	0.81	0.62	0.64	70.8
DNN	0.98	0.81	0.89	0.91	0.56	83.2
1D CNN	0.98	0.86	0.90	0.96	0.91	92.3
1D SNN	0.98	0.97	0.96	0.98	0.99	97.6

**Table 13:** Comparison of precision, recall, and F1 score between the proposed method and the existing methods for each respiration pattern.

Method	Precision	Recall	F1 Score
XGBoost	0.801	0.801	0.801
Decision tree	0.567	0.559	0.563
SVM	0.718	0.701	0.709
RNN	0.798	0.794	0.796
LSTM	0.781	0.775	0.778
GRU	0.707	0.707	0.707
DNN	0.846	0.83	0.838
1D CNN	0.924	0.923	0.923
1D SNN	0.977	0.977	0.977

**Table 14:** Average IOU accuracy of the proposed method and the existing methods for test data.

No.	1D SNN + MASRP (Proposed Method)	1D CNN + MASRP	DNN + MASRP
1	93.1%	81.4%	72.8%
2	93.6%	83.9%	76.4%
3	92.8%	78.6%	71.4%
4	95.2%	84.7%	78.9%
5	94.7%	83.4%	71.8%
…			
49	93.4%	80.2%	73.6%
50	92.5%	79.8%	70.4%
Avg.	93.9%	81.5%	74.2%

## Data Availability

Not applicable.
